# Association Between Peripheral IL‐6 Levels in the Acute Stage of Stroke and Poststroke Depression: A Systematic Review and Meta‐Analysis

**DOI:** 10.1002/brb3.71207

**Published:** 2026-02-09

**Authors:** Hongmin Gong, Jiaqin Yuan, Min Li, Deqi Xiong, Fayang Ling, Mei Liu, Yan Hu, Shouqiang Wang

**Affiliations:** ^1^ Center of Rehabilitation Medicine The Second People's Hospital of Yibin Yibin Sichuan China

**Keywords:** IL‐6, meta‐analysis, poststroke depression, stroke

## Abstract

**Background:**

Interleukin‐6 (IL‐6) has been reported to be associated with depression; however, whether higher peripheral levels of IL‐6 are associated with poststroke depression (PSD) remains controversial. To date, correlative meta‐analyses of the relationship between IL‐6 levels and PSD are lacking.

**Methods:**

We performed a comprehensive search of databases to explore qualified studies reporting IL‐6 levels in the acute phase of stroke and PSD before November 2024. The standard mean deviation (SMD) and 95% confidence interval (CI) were calculated to detect differences in peripheral IL‐6 concentrations between PSD patients and non‐PSD patients.

**Results:**

A total of 22 studies including 4928 participants were included in this meta‐analysis. The results revealed that PSD patients had significantly higher peripheral IL‐6 levels in the acute phase of stroke than non‐PSD patients did (SMD = 0.66, 95% CI = 0.42–0.90). Higher IL‐6 levels were detected in patients with PSD than in non‐PSD patients whether the assessment of depressive symptoms was conducted within 3 months or later, but not at the time of discharge (at discharge: SMD = 1.76, 95% CI: −0.42–3.94, *p* = 0.11; ≤ 3 months: SMD = 2.81, 95% CI: 1.50–4.12, *p* < 0.001; > 3 months: SMD = 3.17, 95% CI: 0.62–5.71, *p* < 0.05). The result of serum for measuring peripheral IL‐6 concentration was significant (SMD = 3.17, 95% CI = [1.63, 4.72], *p* < 0.001); however, plasma was not (SMD = 3.14, 95% CI = [−0.13, 6.40], *p* = 0.06). In addition, HAMD seemed to be more suitable for evaluating depressive symptoms than BDI‐FS (HAMD: SMD = 3.31, 95% CI = [1.86, 4.75], *p* < 0.001; BDI‐FS: SMD = 1.22, 95% CI = [−0.18, 2.62], *p* = 0.09). The sample collection time was the source of high heterogeneity (the subgroup of sample collection time within 1 day: *I* ^2^ = 17%, *p* < 0.001).

**Conclusion:**

Higher peripheral IL‐6 concentrations in the acute stage of stroke are closely related to the risk of PSD; collecting samples within 1 day after stroke onset and evaluating depression post discharge are recommended.

## Graphical abstract

This is a meta‐analysis on the relationship between IL‐6 concentration in the acute phase of stroke and poststroke depression. Higher peripheral IL‐6 concentrations in the acute stage of stroke are closely related to the risk of PSD; collecting samples within 1 day after stroke onset and evaluating depression post discharge are recommended.

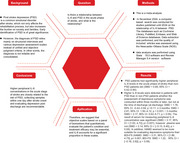

## 1 Introduction

Stroke is one of the most common and harmful cerebrovascular diseases and is accompanied by high disability and mortality rates (Zhou et al. 2024). Furthermore, other critical neurological deficits, including poststroke depression (PSD), dysfunction, dysphagia, and cognitive impairment, also cause serious adverse prognoses (Lim et al. 2021). PSD is a complex emotional problem that occurs in individuals after stroke, and its global occurrence, ranging from 43.3% to 80%, has attracted further research interests of clinicians (Mohammed et al. 2023; Ezema et al. 2019). Patients diagnosed with PSD usually experience sustained emotional decline, slowness of thought, decreased interest, and even suicidal ideation within the first 3 months after stroke (Guo et al. 2022; Lanctôt et al. 2020; Zhou et al. 2024). During the stroke rehabilitation process, depressive symptoms reduce patients’ cooperation, weakening the rehabilitation effect, resulting in poor functional outcomes, and even triggering suicide (Bello et al. 2021; Zemed et al. 2021). In addition, PSD imposes severe economic and mental burdens on the families of patients with stroke and society. Above, predicting and identifying PSD in the early stage of stroke and, in particular, providing therapeutic interventions when stroke survivors experience PSD symptoms are crucial. However, the diagnosis of PSD relies mainly on structured interviews and various depression assessment scales instead of unified and objective judgment criteria. In other words, the diagnosis is not reliable and consolidated.

The pathological mechanisms of PSD are diverse, among which neuroinflammation plays a crucial role in its onset and progression. Currently, research has indicated that inflammatory mechanisms involving hypothalamic–pituitary–adrenal (HPA) axis dysfunction, brain‐derived neurotrophic factor reduction, neurotransmitter and receptor alterations, and diminished neuroplasticity may be breakthrough points for enhancing the objectivity of diagnosing PSD (Frank et al. 2022). The abovementioned connected pathways interact intricately during the neurobiological pathogenesis process instead of working independently, and the effect of inflammatory cytokines is indispensable (L. Wen et al. 2024; Fang et al. 2019). As a pleiotropic cytokine, research evidence has suggested that increased interleukin‐6 (IL‐6) levels in the peripheral or central system play an important role in the pathological mechanisms of depression, especially in HPA axis dysfunction and abnormal neurotransmitter metabolism (Ting et al. 2020). The relationship between IL‐6 levels and decreased appetite, sleep disorders, low mood, and a sense of worthlessness has been confirmed. The key role of IL‐6 in intricate neurobiological mechanisms indicates that IL‐6 is highly important in the diagnosis of depression and in the effect of treatment (Foley et al. 2024). Furthermore, IL‐6 is a small nonstructural protein that is involved in the peripheral inflammatory system and the central inflammatory system and is sensitive for laboratory examinations (Ting et al. 2020). The indispensability of the mechanism and biological characteristics of IL‐6 strongly underscores its potential as an objective biological indicator for diagnosing PSD. However, meta‐analytic evidence is lacking. Therefore, the purpose of this meta‐analysis was to determine the relationship between the level of circulating IL‐6 and PSD in patients and to provide evidence for IL‐6‐related mechanisms and corresponding innovative treatment strategies.

## 2 Review: The Pathology of IL‐6 Impacts on PSD

IL‐6 was first reported in 1986 and considered a B cell stimulator that promotes effector B cells to differentiate into antibody‐producing cells (Hirano et al. 1985). At present, IL‐6 is believed to be a member of the interleukin family, which consists of signaling proteins that promote interactions with immune and nonimmune cells (Khan et al. 2023). In the past, scholars believed that interleukins were only expressed by white blood cells. In fact, monocytes and macrophages are the main producers of IL‐6 in peripheral, and T cells, B cells, liver cells, endothelial cells, fibroblasts, and other cancer cells also produce IL‐6. While in the central system, IL‐6 is mainly secreted by microglia (Akdis et al. 2011). IL‐6 is crucial in the activation, differentiation, proliferation, maturation, migration, and adhesion of immune cells. Its main function is to control cell proliferation, differentiation, and activation in immune and inflammatory responses. Evidence has shown its relationship with the pathogenesis of autoimmune and inflammatory diseases such as psoriasis, inflammatory bowel disease, rheumatoid arthritis, etc. (Khan et al. 2023). As a pro‐inflammatory cytokine, IL‐6 also has been found in the pathogenesis of neurological diseases associated with neuroinflammation due to its vital ability to promote neuroinflammation and mediate blood‐brain barrier disruption by activating the Janus kinase/signal transducer and activator of transcription signaling pathway (Khan et al. 2023). However, due to the different signaling pathways and the involvement of gp130, IL‐6 is of pleiotropy in the immune system. Through the construction of complexes, where IL‐6 binds to the transmembrane IL‐6 receptor (mIL‐6R) or soluble form mediated IL‐6R (sIL‐6R), as well as to the signal transduction subunit molecule gp130, signal transduction of IL‐6 is achieved. IL‐6 has two signaling pathways, the transcription pathway (sIL‐6R) and classical signaling (mIL‐6R), resulting in pro‐inflammatory effects and anti‐inflammatory effects, respectively (Rothaug et al. 2016). Recently, a third signaling pattern called IL‐6 trans‐presentation was discovered, which is mainly through IL‐6R combining to gp130 on nearby located cells (Heink et al. 2017). However, this pattern exists in mice models, not in humans. The dysregulation of the two IL‐6 signaling pathways and excessive production of IL‐6 ultimately lead to depression, neuroinflammation, autoimmune diseases, and the development of cancer, indicating IL‐6 is a master interleukin in the human cytokine network (Uciechowski and Dempke 2020).

Following a stroke, microglia differentiate into M1 and M2 phenotypes, and astrocytes differentiate into the A2 type. Activated M1 microglia release high concentrations of IL‐6, which can increase the synthesis of indoleamine 2,3‐dioxygenase and promote the transformation of astrocytes into A1 type (Qin et al. 2018). Meanwhile, monocytes, macrophages, and endothelial cells in the peripheral system accelerate the secretion of IL‐6 after the occurrence of stroke. During the process of neuroinflammation, IL‐6 levels in peripheral are up to 100 ng/mL, which is 20 times higher than that in healthy individuals (Rose‐John 2017). As a result of the stroke‐induced blood‐brain barrier integrity breakdown, IL‐6 in peripheral blood enters into brain, and triggers further immune cell infiltration and microglial activation. On the other hand, elevated IL‐6 acts on the HPA axis, triggering positive feedback regulation, leading a rise of glucocorticoid and overexpression of tryptophan‐2,3‐dioxygenase (Nagy et al. 2020). Under the action of indoleamine 2,3‐dioxygenase and tryptophan‐2,3‐dioxygenase, L‐tryptophan cannot be converted to 5‐hydroxytryptamine, but to kynurenine and quinolinic acid. The sharp reduction of 5‐hydroxytryptamine in the temporal cortex and left frontal cortex ultimately contributes to PSD (Fang et al. 2019; L. Wen et al. 2024; H. Wen et al. 2018; Feng et al. 2024). And the binding of quinolinic acid and reactive oxygen species reduces the expression of brain‐derived neurotrophic factors, but activates *N*‐methyl‐D‐aspartate receptors. As brain‐derived neurotrophic factors play a key role in regulating depressive symptoms in brain regions by activating pathways related to neurogenesis and synaptogenesis, their reduction is closely related to PSD (R. Hao et al. 2017). Kynurenic acid, which is converted from kynurenine in astrocytes, also prompts the function of *N*‐methyl‐D‐aspartate receptor. The excitability of *N*‐methyl‐D‐aspartate receptors leads an excessive release of glutamate, and its excitotoxicity is associated with decreased synaptic plasticity and increased nerve cell death, ultimately contributing to the onset of PSD (Fang et al. 2019; L. Wen et al. 2024; H. Wen et al. 2018; Feng et al. 2024). In addition, IL‐6 can also directly activate astrocytes to release glutamate in excess and participate in the formation of PSD. Except for 5‐hydroxytryptamine, the deficiency of other monoamine neurotransmitters, dopamine and norepinephrine, is also a significant factor in the development of PSD. Dopamine is synthesized from tyrosine, with tetrahydrobiopterin as a key cofactor. IL‐6‐induced oxidative stress prompts tetrahydrobiopterin convert to dihydrobiopterin, thereby reducing dopamine synthesis. With the decreasing synthesis of dopamine, the effect of converting dopamine to norepinephrine by dopamine β‐hydroxylase weakens, finally reducing synthesis of norepinephrine (Feng et al. 2024). The reduced production of dopamine and norepinephrine ultimately leads to PSD. In summary, the immune inflammatory mechanisms of PSD interact intricately, and IL‐6 is indispensable in these complex mechanisms.

## 3 Materials and Methods

The Preferred Reporting Items for Systematic reviews and Meta‐Analyses (PRISMA) guidelines provided clues for conducting this meta‐analysis (Liberati et al. 2009).

### 3.1 Search Strategy

The studies included in the systematic analysis were retrieved from searches of the Cochrane Library, PubMed, Embase, and Web of Science databases. The retrieval time was set to be from database establishment to November 2024. The search strategy was as follows: (“interleukin‐6” OR “IL‐6” OR “inflammatory marker” OR “Inflammatory Factor”) AND (“poststroke of depression” OR “depression after stroke” OR “poststroke depression” OR “poststroke depression” OR “poststroke depression” OR “PSD”).

### 3.2 Selection Criteria

The inclusion criteria were as follows: (1) the diagnosis of stroke, including ischemic stroke and hemorrhagic stroke, conformed to the criteria of the World Health Organization; (2) patients were admitted to the hospital within 1 month of onset; (3) patients with PSD were evaluated using professional depression scales; (4) all participants were adults; (5) the study type was a cohort or case‐control study; (6) data could be extracted; and (7) the articles were written in English.

The exclusion criteria were as follows: (1) datasets were repeatedly published; (2) studies without available data; (3) reviews, letters, comments, case reports, abstracts, protocols, and conference summaries; and (4) studies not published in English.

### 3.3 Data Extraction

First, duplicate articles were independently removed by the two researchers (Deqi Xiong and Min Li), who browsed the article titles, abstracts, and keywords. Second, the two researchers carefully read the full text after preliminarily browsing the articles and selected articles that met the inclusion and exclusion criteria. Moreover, the first author, publication year, country, sample size, sex differences, age, stroke etiology, time points of the depression assessment, sample collection time, IL‐6 levels, and other essential elements were extracted from the included studies. Because some factors were presented as medians and quartile values, we used a standard method to estimate the means and standard deviations (Hozo et al. 2005). In the above process, any disagreement was resolved by consulting a third expert (Mei Liu).

### 3.4 Quality Assessment

The methodological quality of the research included in the meta‐analysis was independently evaluated by two researchers (Fayang Ling and Yan Hu) using the Newcastle–Ottawa Scale (NOS). The NOS, with a maximum score of 9 stars, is divided into three sections: subject selection (4 items), intergroup comparability (1 item), and outcome (3 items).

### 3.5 Statistical Analysis

We used Stata 18.0 (StataCorp LP, College Station) to calculate the standardized mean difference (SMD) and confidence interval (CI) of IL‐6 concentrations in survivors with PSD and non‐PSD patients to estimate the relationship between IL‐6 levels and PSD. The heterogeneity between different articles was tested by *I* ^2^, of which 25%, 50%, and 75% were considered low, moderate, and high heterogeneity, respectively. If the heterogeneity was moderate or high (*p* < 0.05, *I* ^2^ > 50.0%), a random‐effects model was selected for analysis. Otherwise, the fixed‐effects model was chosen. Subgroup analyses were conducted using Review Manager software(version 5.4, Cochrane Collaboration, London, UK), and based on race, stroke type, sample sources, depression assessment scale scores, blood collection times, time points of the depression assessment, and study quality to identify the sources of heterogeneity. Moreover, meta‐regression analyses were performed to explore the sources of heterogeneity in depth, and the covariates were the proportion of females, average age, body mass index (BMI), National Institutes of Health Stroke Scale (NIHSS) score, and years of education. Funnel plots and Egger's test were used to assess publication bias.

## 4 Results

### 4.1 Literature Selection

**FIGURE 1:**
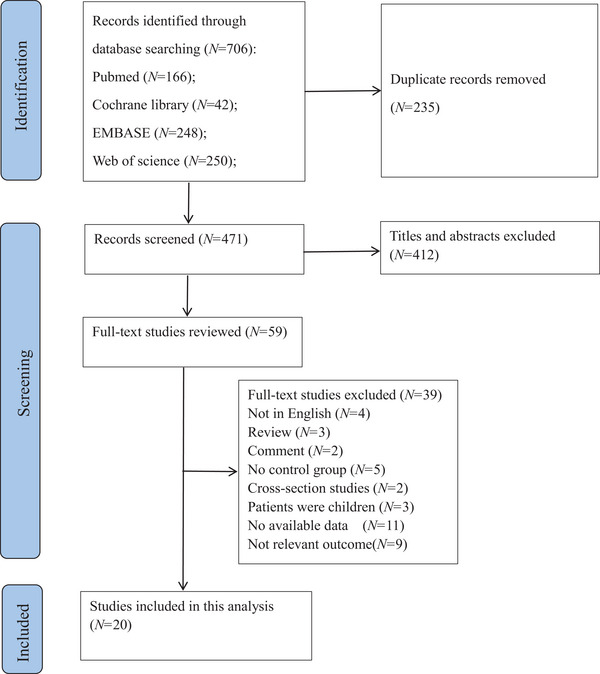
Flowchart of the study selection process.

A total of 706 articles related to the level of IL‐6 in patients with depression after acute stroke were initially identified via the search strategy. A total of 235 duplicates were removed first, and 412 articles were then deleted after the preliminary and rough screening of the titles and abstracts. At this stage, 59 articles were eligible for full‐text retrieval, 39 of which were excluded based on the inclusion and exclusion criteria. Finally, 20 original articles were included (Figure 1). Due to the fact that serum samples of IL‐6 were collected both on the days 2 and 8 after stroke in the article by Yang et al. 2010, we used Yang (a) and Yang (b) to distinguish them respectively. And Korostynski et al. (2021) evaluated depressive symptoms on the 8th day and 3 months after stroke (29), Korostynski (a) and Korostynski (b) were used for differention in this study. Thereby, 20 articles were included in this meta‐analysis, but actually 22 results were shown.

### 4.2 Characteristics and Quality Assessment

A total of 4928 participants, of whom 1665 patients were in the PSD group, and another 3263 patients were in the non‐PSD group, were included in the meta‐analysis (Table 1). The basic information, including the first author and publication year, country, and other elements of the 22 included studies, is shown in Table 1. Notably, the studies included were generally of high quality, with NOS scores of 6–9 (Table 1).

**TABLE 1:** Main characteristics of the included studies.

	Number (M/F)		Age (years)		NIHSS score		Study design	Country	Type of stroke	Follow‐up	Sample collection time	Sample source	NOS score
	PSD NPSD		PSD NPSD		PSD NPSD								
Yang (a) et al. 2010	18/19	37/26	68.95 ± 9.28	68.43 ± 11.18	6.82 ± 4.24	3 ± 1.52	Cohort study	China	AIS	2 w	7 d	Serum	7
Yang (b) et al. 2010	18/19	37/26	68.95 ± 9.28	68.4 3± 11.18	6.82 ± 4.24	3 ± 1.52	Cohort study	China	AIS	2 w	1 d	Serum	7
Su et al. 2012	—	—	—	—	—	—	Cohort study	China	Acute stroke	1 y	In the hospital	Serum	7
Spalletta et al. 2013	—	—	—	—	—	—	Case‐control study	Italy	First‐ever stroke	6 d	3 d	Serum	6
Jiao et al. 2016	51/31	154/119	—	—	—	—	Cohort study	China	First‐ever AIS	2 y	In the hospital	Serum	6
H. J. Kang et al. 2016	22/31	124/82	66 ± 9.3	63.9 ± 9.5	—	—	Cohort study	Korea	AIS	1 y	2 w	Serum	7
Zhang et al. 2016	19/15	35/31	62.4± 6.2	64.1 ± 5.1	—	—	Cohort study	China	First‐ever stroke	3 w	1 w	Serum	8
Meng et al. 2017	22/14	21/26	67.9 ± 9.1	69.8 ± 10.4	3.36 ± 3.86	1 ± 1.53	Cohort study	China	AIS	1 w	1 d	Serum	7
Y. Wang et al. 2022	24/14	19/28	70.17 ± 10.786	68.66 ± 9.607	—	—	Cohort study	China	First‐ever AIS	10 d	1 d	Serum	7
Q. Wang et al. 2018	27/18	68/39	62.34 ± 14.17	64.65 ± 9.77	3.18 ± 1.91	2 ± 1.5	Cohort study	China	Acute stroke	1 m	4 d	Serum	7
Mu et al. 2018	25/35	28/32	56.2 ± 13.2	54.4 ± 11.2	—	—	Case‐control study	China	First‐ever stroke	In the hospital	In the hospital	Serum	6
Xu et al. 2018	52/43	238	—	—	8.70 ± 6.02	—	Case‐control study	China	First‐ever AIS	3 m	—	Plasma	7
G. Li et al. 2020	173/59	60/206	58.34 ± 10.49	56.14 ± 11.13	5 ± 4.48	2.35 ± 2.24	Cohort study	China	First‐ever stroke	At discharge	8 d	Serum	8
Lu et al. 2020	32/44	172/62	62.71 ± 15.11	62 ± 16.41	11.35 ± 3.78	7.35 ± 5.22	Cohort study	China	First‐ever stroke	3 m	3 d	Serum	9
Dai et al. 2021	34/12	97/42	65.74 ± 10.17	62.95 ± 11.34	—	—	Cohort study	China	First‐ever AIS	6 m	—	Serum	8
Y. Kang et al. 2021	—	—	—	—	—	—	Cohort study	China	Ischemic stroke	—	2–4 w	Serum	6
Korostynski (a) et al. 2021	7/20	84/59	65.93 ± 11.74	68.76 ± 12.73	8.07 ± 7.04	8.76 ± 6.74	Cohort study	Poland	AIS	8 d	1 d	Plasma	7
Korostynski (b) et al. 2021	11/26	64/45	65.64 ± 11.57	66.35 ± 14.27	9 ± 4.63	8.41 ± 7.51	Cohort study	Poland	AIS	3 m	1 d	Plasma	8
G. Li et al. 2021	192/57	211/58	60.65 ± 9.69	58.3 ± 10.43	7.65 ± 3.73	2.35 ± 2.24	Cohort study	China	First‐ever stroke	At discharge	8 d	Serum	9
Y. Wang et al. 2021	170/66	338/82	59.65 ± 11.19	58.35 ± 11.16	4.05 ± 3.73	2.70 ± 2.98	Cohort study	China	First‐ever stroke	6 m	—	Serum	8
Y. Li, Xie et al. 2022	—	—	—	—	—	—	Cohort study	China	AIS	At discharge	In the hospital	Serum	6
L. Wang et al. 2023	63/28	147/61	65.6 ± 15.3	63.1 ± 14.3	—	9.9 ± 4.9	Cohort study	China	first‐ever stroke	At discharge	In the hospital	Plasma	6

Abbreviations: AIS, acute ischemic stroke; d, day; F, female; M, male; m, month; NIHSS, National Institutes of Health Stroke Scale; NOS, Newcastle–Ottawa Scale; NPSD, non‐poststroke depression; w, week; y, year.

### 4.3 IL‐6 Levels

#### 4.3.1 Meta‐Analysis

**FIGURE 2:**
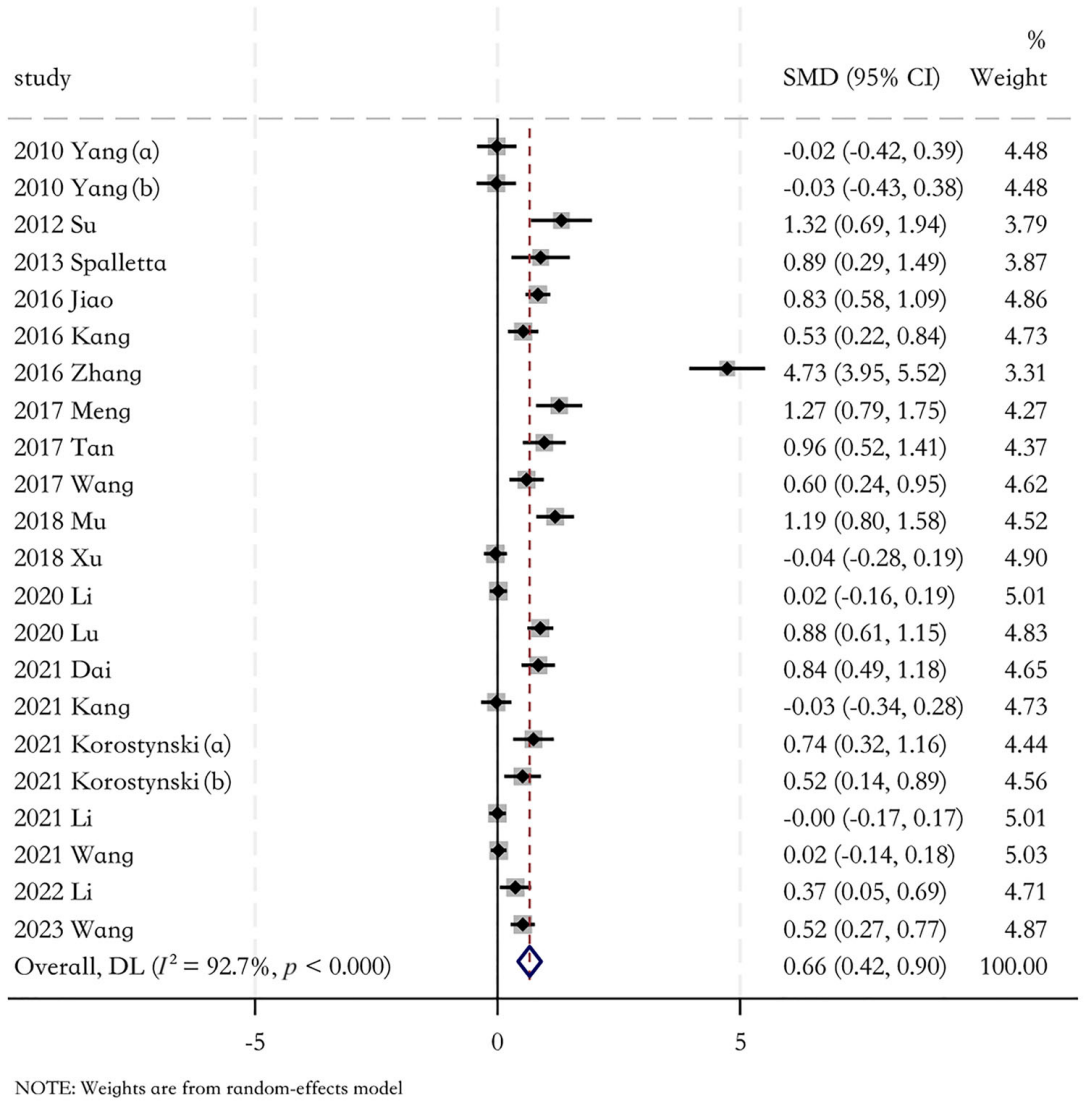
Results of meta‐analysis on IL‐6 levels in poststroke depression (PSD) patients and the non‐PSD.

The heterogeneity was obvious (*I* ^2^ = 92.7%, *p* < 0.001). A random‐effects model was used to analyze the differences in IL‐6 levels between PSD patients and the controls. The combined value revealed that IL‐6 levels in patients with PSD were significantly higher than those in controls (SMD = 0.66, 95% CI = 0.42–0.90) (Figure 2).

#### 4.3.2 Sensitivity Analysis

**FIGURE 3:**
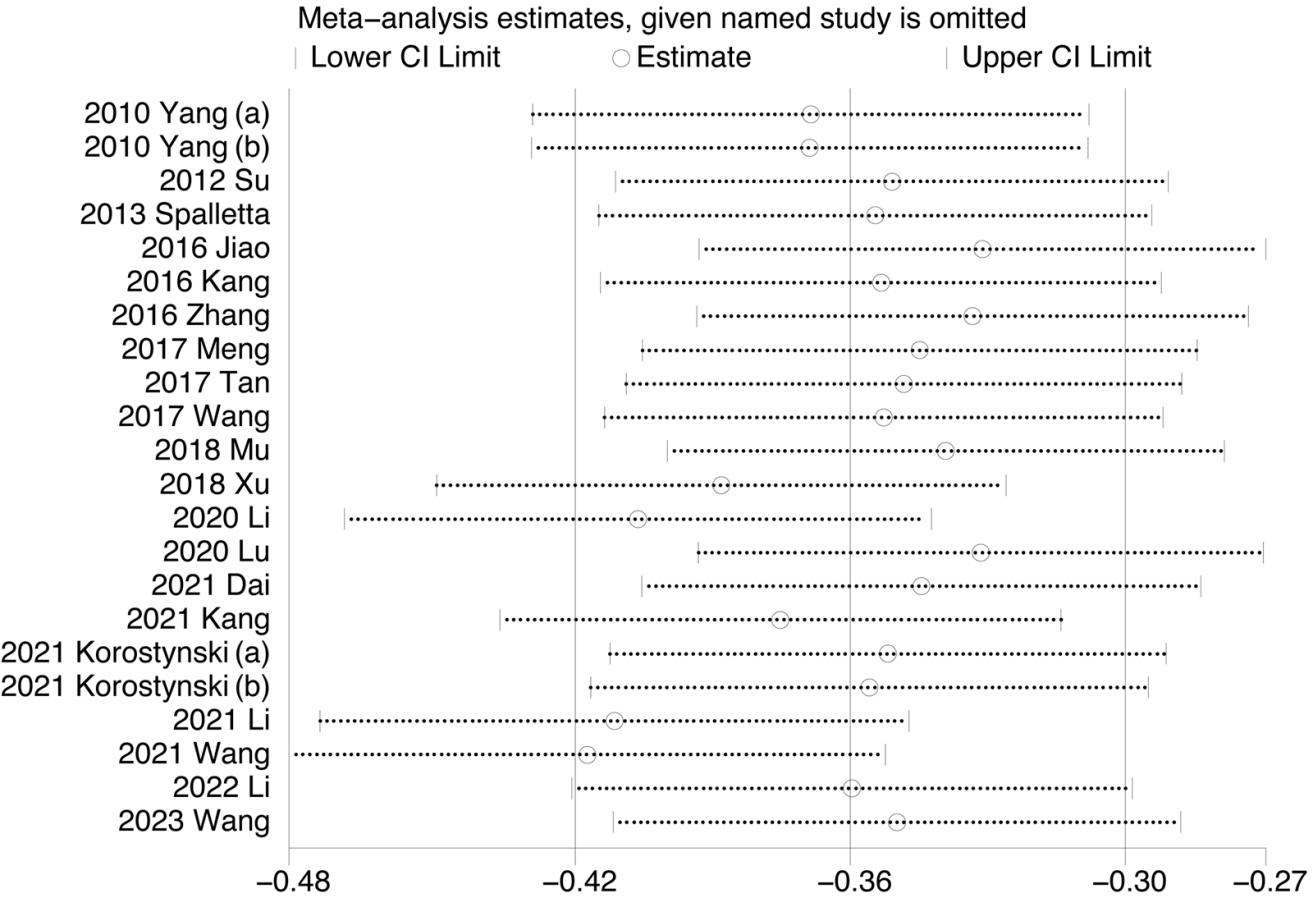
The sensitivity analysis for the meta‐analysis.

The results revealed that no study had a prominent effect on the comprehensive effect of the main outcome, indicating that the outcome was stable (Figure 3).

#### 4.3.3 Subgroup Analysis

We conducted seven subgroup analyses based on the differences in race, type of stroke, depression assessment scale, depression assessment time, sample collection time, sample sources, and the NOS score (Table 2). The heterogeneity of IL‐6 in the European subgroup was small (*I* ^2^ = 0%, *p* = 0.44; Table 2). When analyzing 11 articles that only included acute ischemic stroke (AIS) patients, the upregulation of IL‐6 levels was still significant (SMD = 0.85, 95% CI = [0.62, 1.07], *p* < 0.001). Based on the subgroup analysis of the depression assessment scale, IL‐6 concentrations were obviously elevated in patients with PSD according to the Hamilton Depression (HAMD) scale (SMD = 3.31, 95% CI = [1.86, 4.75], *p* < 0.001) but not in those assessed with the Beck Depression Inventory‐Fast Screen (BDI‐FS) (SMD = 1.22, 95% CI = [−0.18, 2.62], *p* = 0.09). Higher IL‐6 levels were detected in patients with PSD than in non‐PSD patients whether the assessment of depressive symptoms was conducted within 3 months or later, but not at the time of discharge (at discharge: SMD = 1.76, 95% CI: −0.42–3.94, *p* = 0.11; ≤ 3 months: SMD = 2.81, 95% CI: 1.50–4.12, *p* < 0.001;> 3 months: SMD = 3.17, 95% CI: 0.62–5.71, *p* < 0.05). Notably, we found that the association between higher IL‐6 levels and the incidence of PSD was more significant when blood samples were collected within 1 day after stroke (SMD = 1.34, 95% CI = [0.89, 1.78], *p* < 0.001), with substantially reduced heterogeneity (*I* ^2^ = 17%, *p* < 0.001). In addition, the result of serum for measuring peripheral IL‐6 concentration was significant (SMD = 3.17, 95% CI = [1.63, 4.72], *p* < 0.001); however, plasma was not (SMD = 3.14, 95% CI = [−0.13, 6.40], *p* = 0.06). In high‐quality studies, the heterogeneity of IL‐6 levels was slightly reduced (*I* ^2^ = 81%, *p* < 0.001), and the significance was not modifiable (SMD = 0.76, 95% CI = [0.56, 0.95], *p* < 0.001) (Table 2).

**TABLE 2:** Comparison of IL‐6 levels between PSD and non‐PSD in different subgroups.

Variable	Sample Characteristic	Number of studies	Heterogeneity test		SMD (95% CI)	*p* value
			*p* value	*I* ^2^ value (%)		
IL‐6						
	Race					
	European	3	0.44	0	2.93 (0.77, 5.10)	0.008
	Asian	19	< 0.001	96	2.84 (1.75, 3.94)	< 0.001
	Type of stroke					
	AIS	11	< 0.001	85	0.85 (0.62, 1.07)	< 0.001
	Stroke	10	< 0.001	97	2.04 (1.71, 2.38)	< 0.001
	Type of depression assessment scale					
	HAMD	16	< 0.001	95	3.31 (1.86, 4.75)	< 0.001
	BDI‐FS	3	0.09	95	1.22 (−0.18, 2.62)	0.09
	Depression assessment time					
	≤ 3 months after stroke	12	< 0.001	96	2.81 (1.50, 4.12)	< 0.001
	> 3 months after stroke	5	0.01	87	3.17 (0.62, 5.71)	0.01
	At discharge	4	0.11	82	1.76 (−0.42, 3.94)	0.11
	Sample collection time					
	In the hospital	5	< 0.001	84	5.62 (2.18, 9.06)	0.001
	≤ 1 day after stroke	5	0.3	17	1.13 (0.74, 1.52)	< 0.001
	> 1 day after stroke	8	< 0.001	97	3.30 (0.79, 5.80)	0.01
	Sample resource					
	Serum	18	< 0.001	98	3.17 (1.63, 4.72)	< 0.001
	Plasma	4	< 0.001	86	3.14 (−0.13, 6.40)	0.06
	NOS score					
	< 7 scores	7	< 0.001	99	1.59 (1.17, 2.01)	< 0.001
	≥ 7 scores	15	< 0.001	81	0.76 (0.56, 0.95)	< 0.001

Abbreviations: AIS, acute ischemic stroke; BDI‐FS, Beck Depression Inventory‐Fast Screen; NIHSS, National Institutes of Health Stroke Scale; NOS, Newcastle–Ottawa Scale.

#### 4.3.4 Bias Analysis

**FIGURE 4:**
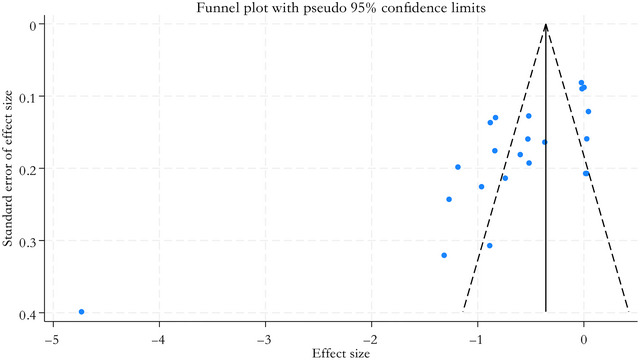
Funnel plot showing the impact of elevated IL‐6 level on poststroke depression.

The funnel plot was visually asymmetric, and more points were distributed on the left. The results of Egger's test were significant (*p* = 0.000), suggesting potential publication bias (Figure 4). After further exploration using the trim‐and‐fill method, we obtained similar results.

#### 4.3.5 Regression Analyses

The meta‐regression analysis revealed that age, sex, BMI, NIHSS score, and years of education did not account for the high degree of heterogeneity (Table 3).

**TABLE 3:** Results of meta‐regression.

Index	Variable	Coefficients	95% CI	*p* value
IL‐6	Rate of female sex	−0.474	−2.003–1.056	0.544
	Mean age	−1.826	−10.318–6.667	0.674
	BMI	−0.999	−4.602–2.604	0.587
	NIHSS score	−0.319	−1.122–0.484	0.436
	Education year	0.545	−1.433–2.522	0.589

## 5 Discussion

### 5.1 Explanation of Results

The incidence of PSD in this study was 33.79%, which was similar to previous studies (Chen et al. 2022). The results from 22 studies including 4928 participants revealed that, in the acute stroke stage (we define the first month after stroke as the acute phase of stroke according to previous researches [R. Liu, Liu et al. 2023; F. Liu et al. 2024]), the IL‐6 levels of patients with PSD were significantly higher than those in the non‐PSD group. These findings suggest that, in terms of PSD, IL‐6 may be a potential biomarker for diagnosis and a parameter predicting response to treatment of patients in the acute phase of stroke.

A key finding to emerge from the current analyses was the positive association between PSD and IL‐6 levels in peripheral blood, which was consistent with the conclusions of published research (Chen et al. 2020; Zheng et al. 2024). As a master regulator in the inflammation network, IL‐6 levels in the peripheral blood have been reported to be closely associated with other types of depression (Almeida et al. 2020; Vöckel et al. 2024; Jara et al. 2020). In animal models of depression‐like behavior, peripheral and hippocampal levels of IL‐6 are simultaneously increased after stress stimulation (Monje et al. 2011). These studies support the hypothesis that peripheral IL‐6 levels may intuitively reflect changes in the central nervous system. However, the hypothesis is in contrast to the findings, that lower IL‐6 levels in hippocampus of rat may increase the risk of depressive behavior, and its overexpression in the CA1 region rescued the neurological transformation and depressive behaviors (P. Wang et al. 2019; Young et al. 2014). These contradictory findings may suggest a more complex relationship between IL‐6 levels in the periphery and cerebrospinal fluid, where IL‐6 is released in a neuroprotective manner after stress, or they may indicate that the role of IL‐6 in animal depression models is different from that in humans with depression.

In this study, the value of heterogeneity was not small and should not be easily ignored. Fortunately, we identified the source of heterogeneity. This value was significantly reduced in the subgroup analysis that included articles in which the blood collection time was within 1 day from stroke onset. In a study of 175 AIS patients, IL‐6 was detected by enzyme‐linked immunosorbent assay (ELISA) at baseline and on Day (D) 1, D3, and D7 after admission, the results showed that IL‐6 levels increased from baseline to D1 and then gradually decreased until D7 (Shi et al. 2023). Furthermore, a growing body of evidence has confirmed that elevated IL‐6 concentrations on the first day after stroke are related to the deterioration of neurological function and a wider range of ischemic lesions (Bustamante et al. 2014; Martínez‐Sánchez et al. 2014; Hotter et al. 2019). Hanna et al. reported that the level of IL‐6 detected within 24 h could be used to statistically assess the long‐term prognosis of stroke patients, even 1 year after stroke, whereas the level of IL‐6 at D7 could not (Shi et al. 2023). This finding was inconsistent with previous analyses, which revealed that 7 days after stroke occurrence may be the optimal blood sampling time for predicting PSD (Chen et al. 2020). Therefore, we speculate that IL‐6 levels can be used to predict the long‐term prognosis of PSD patients, but the effect may be limited to a specific time window. In another study, when blood samples were collected within 72 h after stroke onset, no obvious association between IL‐6 levels and the development of PSD was identified beyond 6 months (Ormstad et al. 2012). This indistinguishable result may be strongly associated with the different time points of blood collection. Together, the above findings indicate that clinical or fundamental investigations with unified blood collection times may be needed in the future and that earlier blood sampling after stroke may be of greater clinical significance.

The sensitivity analyses revealed that the primary result was not significant when IL‐6 levels were measured in plasma samples, suggesting that the serum levels of IL‐6 may be more suitable for predicting the occurrence and development of PSD (Köhler et al. 2017). Both plasma and serum are sensitive to variations in the internal and external environments of the human body and can provide vital information at the system level. Serum seems to have a more robust effect on the stability of human metabolites or lipoproteins, and its composition results from clotting only by centrifugation, whereas plasma samples are obtained by adding anticoagulants that partially inhibit plasma protein hydrolytic activity (Vignoli et al. 2022; Yi et al. 2007). Another study revealed that, compared with other types of detection, ELISA detection of IL‐6 levels has greater heterogeneity (Köhler et al. 2017). However, all samples in our meta‐analysis were tested using ELISA, and thus we did not explore the impact of differences in testing methods on the results.

In addition, we found differences in the validity of HAMD and BDI‐FS in evaluating PSD, and HAMD seems to be more effective. HAMD was first proposed by British psychiatrist Max Hamilton in 1960 and is considered the “gold standard” for assessing the severity of depression in clinical trials of antidepressant treatment (HAMILTON 1960). As a type of clinician‐rated scale, HAMD is evaluated by professionals combined with observation and interviews, reducing subjective bias and increasing the reliability of results. In contrast, BDI‐FS is a part of self‐rating scales with stronger subjectivity. And the drawback of containing items that may be difficult to understand narrows down the applicable population (X. Li et al. 2025). Recently, a meta‐analysis comparing nine PSD assessment scales, including Patient Health Questionnaire‐9 (PHQ‐9), The Patient Health Questionnaire‐2 (PHQ‐2), Montgomery Åsberg Depression Rating Scale (MADRS), HAMD scale, Hospital Anxiety and Depression Scale‐Depression (HADS‐D), Geriatric Depression Screening Scale (GDS), Center for Epidemiological Studies‐Depression (CES‐D), Beck Depression Inventory (BDI), showed that HAMD and PHQ‐9 had higher diagnostic accuracy in assessing depressive symptoms during both the acute and chronic phases of stroke (F. Liu et al. 2024). In the chronic phase of stroke, the sensitivity, specificity, and diagnostic odds ratio of HAMD were 0.94, 0.85, and 96, respectively. And for BDI‐FS, the scores were 0.92, 0.81, and 51 (F. Liu et al. 2024). But in our study, most articles used HAMD for evaluating PSD, and only three articles used BDI‐FS, which may have caused bias in the results.

The relationship between inflammatory factors and depression has long been studied, and the pathophysiological mechanisms involved have proven to be complex (Harsanyi et al. 2022). Although IL‐6, as mentioned in this article, plays a vital role in the inflammatory mechanism of PSD, large‐scale epidemiological studies and meta‐analyses have provided evidence for strong associations between the levels of IL‐1β, IL‐2, IL‐4, IL‐10, IL‐17, IL‐18, IL‐33, and tumor necrosis factor‐alpha (TNF‐α) and PSD (Zheng et al. 2024; Y. Hao et al. 2019; Neupane et al. 2022; Yui et al. 2022; Byrne et al. 2020). It has been reported that IL‐1β, IL‐6, and IL‐17 act as pro‐inflammatory factors in neuroinflammation, and IL‐2, IL‐4, and IL‐10 utilize anti‐inflammatory properties conversely (Khan et al. 2023). Recently, a meta‐analysis of circulating interleukin concentrations and PSD showed that compared with NPSD patients, the peripheral concentrations of IL‐1, IL‐6, and IL‐18 were significantly higher in PSD patients during the acute phase, while the concentration of IL‐10 was lower (Zheng et al. 2024). And IL‐33 is negatively correlated with increased risk of PSD, indicating IL‐33 has a protective effect on depression. The effect may be through the IL‐33/ST3/NF‐KB pathway: IL‐33 promotes microglia polarization toward M2 macrophages, enhances synaptic remodeling, inhibits gamma‐aminobutyric acid conduction, and finally inhibits the occurrence of depression (R. Liu, Liu et al. 2023). In the acute phase of stroke, in addition to secrete pro‐inflammatory cytokines IL‐6, activated microglia also secrete IL‐1β and TNF‐α, which play a role in the pathological process of PSD. A meta‐analysis including 889 acute stroke patients showed that the serum concentrations of IL‐6 and TNF‐α in the PSD group were higher than those in non‐PSD group. However, this analysis only includes 8 original articles, the data analysis is not detailed enough, and the results may be unstable (Chen et al. 2020). In a 2‐year longitudinal study, Y. Li, Xie et al. (2022) used diffusion tensor imaging, IL‐6 level measurement, and standardized clinical scales to evaluate the importance of differences in the IL‐6‐white matter network for the development of depression, finally found that the individual differences in depressive outcomes are caused by variations in the IL‐6‐white matter network, suggesting IL‐6 is reliable in predicting susceptibility to depression. Furthermore, in numerous meta‐analyses and clinical studies, IL‐6 has demonstrated its reliability in depression compared to other inflammatory factors, and it is sensitive to laboratory measurements of acute stress and plays an important role in mood disorders (Dowlati et al. 2010; Goldsmith et al. 2016; Haapakoski et al. 2015). Therefore, we suggest that objective scales based on a panel of biomarkers that quantitatively evaluate the patient's condition and treatment efficacy may be essential, and IL‐6 accounts for a significant proportion in these scales.

### 5.2 Clinical Implications

According to the results of this study, we speculate that IL‐6 is a potential biomarker for diagnosis for PSD, but the effect may be limited to a specific time window and earlier blood sampling after stroke is of greater clinical significance. And reducing peripheral IL‐6 levels through IL‐6 receptor antibodies or IL‐6 antibodies may be a therapeutic strategy. However, the complexity and time uncertainty of the use of peripheral blood IL‐6 levels in assessing the pathogenesis of PSD may change the way in which depression disorders are treated. IL‐6 has two signaling pathways, the transcription pathway and classical signaling, resulting in pro‐inflammatory effects and anti‐inflammatory effects, respectively (Rothaug et al. 2016). Therefore, in addition to simply blocking IL‐6 signaling, appropriately amplifying IL‐6 signaling according to the timeline and unique inflammatory response of each patient may be beneficial for treating PSD. During the process of trans signaling, IL‐6 is bound to a soluble IL‐6 receptor (sIL‐6R) and forms the binary IL‐6:sIL‐6R complex, which has a pro‐inflammatory effect. However, soluble gp130 (sgp130) antagonizes trans signaling by establishing a ternary complex, namely, IL‐6:sIL‐6R:sgp130 (Monsour et al. 2023). Thus, the sgp130 antibody can be used to promote the formation of ternary complexes, thereby enhancing the anti‐inflammatory effect of IL‐6 for the treatment of depression (Morieri et al. 2017). The binary/ternary (B/T) ratio is expected to be used to explore the optimal time for enhancing or inhibiting the trans IL‐6 signaling pathway, and more related research is needed in the future.

### 5.3 Limitations

Notably, our meta‐analysis has several limitations. First, this study excluded studies published in languages other than English, resulting in language limitations. Second, PSD is a psychiatric complication after stroke that may occur at any time within 5 years after stroke. Among the studies we included, the longest follow‐up time was up to 2 years, and the shortest was only 6 days. Future research may require longer and more consistent follow‐up periods. Third, most of the included studies did not clarify the use of antidepressants, and whether antidepressants had an impact on the primary results requires further discussion. Fourth, different scales were used to evaluate depression in included articles, making the comparison results more challenging. A unified evaluation method is needed in the future to improve the reliability. Furthermore, the high heterogeneity and publication bias should not be ignored, and more studies are needed in the future to explore the impact of IL‐6 on PSD.

## 6 Conclusion

In summary, the peripheral blood IL‐6 levels in PSD patients during the acute phase of stroke were significantly higher than those in patients without PSD, indicating that peripheral IL‐6 levels in patients with acute stroke are associated with an increased risk of PSD. This relationship is more stable when collecting serum samples within 1 day after stroke occurrence. Therefore, IL‐6 is an important potential inflammatory factor for predicting PSD, and collecting samples within 1 day after stroke onset and evaluating depression post discharge are recommended.

## Author Contributions

**Hongmin Gong**: Methodology; conceptualization; writing; investigation. **Jiaqin Yuan**: Writing; project administration; investigation; conceptualization. **Min li**: Software; data curation; formal analysis. **Deqi Xiong**: Formal analysis; software; data curation. **Fayang Ling**: Validation; data curation; visualization. **Mei Liu**: Data curation; validation; methodology. **Yan Hu**: All authors have read and approved the final version to be published.

## Funding

This research was funded by the Open Project of Sichuan Key Laboratory of Rehabilitation Medicine (grant no. KFYXSZDSYS‐05).

## Conflicts of Interest

The authors declare no conflicts of interest.

## Data Availability

The original data of this article will be provided by the author and will not be improperly retained.

## References

[brb371207-bib-0001] Akdis, M. , S. Burgler , R. Crameri , et al. 2011. “Interleukins, From 1 to 37, and Interferon‐γ: Receptors, Functions, and Roles in Diseases.” Journal of Allergy and Clinical Immunology 127, no. 3: 701–721.e1–70. 10.1016/j.jaci.2010.11.050. 21377040

[brb371207-bib-0002] Almeida, I. B. , I. A. Gomes , S. Shanmugam , et al. 2020. “Inflammatory Modulation of Fluoxetine Use in Patients With Depression: A Systematic Review and Meta‐Analysis.” Cytokine 131: 155100. 10.1016/j.cyto.2020.155100. 32315957

[brb371207-bib-0003] Bello, U. M. , M. Chutiyami , D. Salihu , et al. 2021. “Quality of Life of Stroke Survivors in Africa: A Systematic Review and Meta‐Analysis.” Quality of Life Research 30, no. 1: 1–19. 10.1007/s11136-020-02591-6. 32712933

[brb371207-bib-0004] Bustamante, A. , T. Sobrino , D. Giralt , et al. 2014. “Prognostic Value of Blood Interleukin‐6 in the Prediction of Functional Outcome After Stroke: A Systematic Review and Meta‐Analysis.” Journal of Neuroimmunology 274, no. 1–2: 215–224. 10.1016/j.jneuroim.2014.07.015. 25091431

[brb371207-bib-0005] Byrne, M. L. , N. M. O'Brien‐Simpson , E. C. Reynolds , et al. 2020. “Corrigendum to ‘Acute Phase Protein and Cytokine Levels in Serum and Saliva: A Comparison of Detectable Levels and Correlations in a Depressed and Healthy Adolescent Sample’ [Brain Behav. Immun. 34 (2013) 164–175].” Brain, Behavior, and Immunity 89: 696–697. 10.1016/j.bbi.2020.07.003. 32694075

[brb371207-bib-0006] Chen, Y. , J. Pu , Y. Liu , et al. 2020. “Pro‐Inflammatory Cytokines Are Associated With the Development of Post‐Stroke Depression in the Acute Stage of Stroke: A Meta‐Analysis.” Topics in Stroke Rehabilitation 27, no. 8: 620–629. 10.1080/10749357.2020.1755813. 32316861

[brb371207-bib-0007] Chen, Y. , H. Zou , M. Peng , and Y. Chen . 2022. “Association Between Homocysteine Levels in Acute Stroke and Poststroke Depression: A Systematic Review and Meta‐Analysis.” Brain and Behavior 12, no. 6: e2626. 10.1002/brb3.2626. 35605010 PMC9226802

[brb371207-bib-0008] Dai, M. , Q. Wei , Y. Zhang , C. Fang , P. Qu , and L. Cao . 2021. “Predictive Value of Red Blood Cell Distribution Width in Poststroke Depression.” Computational and Mathematical Methods in Medicine 2021: 8361504. 10.1155/2021/8361504. 34335867 PMC8315889

[brb371207-bib-0009] Dowlati, Y. , N. Herrmann , W. Swardfager , et al. 2010. “A Meta‐Analysis of Cytokines in Major Depression.” Biological Psychiatry 67, no. 5: 446–457. 10.1016/j.biopsych.2009.09.033. 20015486

[brb371207-bib-0010] Ezema, C. I. , P. C. Akusoba , M. C. Nweke , C. U. Uchewoke , J. Agono , and G. Usoro . 2019. “Influence of Post‐Stroke Depression on Functional Independence in Activities of Daily Living.” Ethiopian Journal of Health Sciences 29, no. 1: 841–846. 10.4314/ejhs.v29i1.5. 30700951 PMC6341441

[brb371207-bib-0011] Fang, M. , L. Zhong , X. Jin , et al. 2019. “Effect of Inflammation on the Process of Stroke Rehabilitation and Poststroke Depression.” Frontiers in Psychiatry 10: 184. 10.3389/fpsyt.2019.00184. 31031649 PMC6470379

[brb371207-bib-0012] Feng, X. , X. Ma , J. Li , et al. 2024. “Inflammatory Pathogenesis of Post‐Stroke Depression.” Aging and Disease 16: 209. 10.14336/AD.2024.0203. 38377025 PMC11745428

[brb371207-bib-0013] Foley, É. M. , C. Slaney , N. A. Donnelly , M. Kaser , L. Ziegler , and G. M. Khandaker . 2024. “A Novel Biomarker of Interleukin 6 Activity and Clinical and Cognitive Outcomes in Depression.” Psychoneuroendocrinology 164: 107008. 10.1016/j.psyneuen.2024.107008. 38442505 PMC7617704

[brb371207-bib-0014] Frank, D. , B. F. Gruenbaum , A. Zlotnik , M. Semyonov , A. Frenkel , and M. Boyko . 2022. “Pathophysiology and Current Drug Treatments for Post‐Stroke Depression: A Review.” International Journal of Molecular Sciences 23, no. 23: 15114. 10.3390/ijms232315114. 36499434 PMC9738261

[brb371207-bib-0015] Goldsmith, D. R. , M. H. Rapaport , and B. J. Miller . 2016. “A Meta‐Analysis of Blood Cytokine Network Alterations in Psychiatric Patients: Comparisons Between Schizophrenia, Bipolar Disorder and Depression.” Molecular Psychiatry 21, no. 12: 1696–1709. 10.1038/mp.2016.3. 26903267 PMC6056174

[brb371207-bib-0016] Guo, J. , J. Wang , W. Sun , and X. Liu . 2022. “The Advances of Post‐Stroke Depression: 2021 Update.” Journal of Neurology 269, no. 3: 1236–1249. 10.1007/s00415-021-10597-4. 34052887

[brb371207-bib-0017] Haapakoski, R. , J. Mathieu , K. P. Ebmeier , H. Alenius , and M. Kivimäki . 2015. “Cumulative Meta‐Analysis of Interleukins 6 and 1β, Tumour Necrosis Factor‐α and C‐Reactive Protein in Patients With Major Depressive Disorder.” Brain, Behavior, and Immunity 49: 206–215. 10.1016/j.bbi.2015.06.001. 26065825 PMC4566946

[brb371207-bib-0018] Hamilton, M. 1960. “A Rating Scale for Depression.” Journal of Neurology, Neurosurgery, and Psychiatry 23: 56–62. 10.1136/jnnp.23.1.56. 14399272 PMC495331

[brb371207-bib-0019] Hao, R. , Y. Qi , D. N. Hou , et al. 2017. “BDNF val66met Polymorphism Impairs Hippocampal Long‐Term Depression by Down‐Regulation of 5‐HT3 Receptors.” Frontiers in Cellular Neuroscience 11: 306. 10.3389/fncel.2017.00306. 29075179 PMC5643500

[brb371207-bib-0020] Hao, Y. , J. Ding , R. Hong , et al. 2019. “Increased Interleukin‐18 Level Contributes to the Development and Severity of Ischemic Stroke.” Aging 11, no. 18: 7457–7472. 10.18632/aging.102253. 31525735 PMC6781996

[brb371207-bib-0021] Harsanyi, S. , I. Kupcova , L. Danisovic , and M. Klein . 2022. “Selected Biomarkers of Depression: What Are the Effects of Cytokines and Inflammation.” International Journal of Molecular Sciences 24, no. 1: 578. 10.3390/ijms24010578. 36614020 PMC9820159

[brb371207-bib-0022] Heink, S. , N. Yogev , C. Garbers , et al. 2017. “Trans‐Presentation of IL‐6 by Dendritic Cells Is Required for the Priming of Pathogenic T(H)17 Cells.” Nature Immunology 18, no. 1: 74–85. 10.1038/ni.3632. 27893700 PMC5164931

[brb371207-bib-0023] Hirano, T. , T. Taga , N. Nakano , et al. 1985. “Purification to Homogeneity and Characterization of Human B‐Cell Differentiation Factor (BCDF or BSFp‐2).” Proceedings of the National Academy of Sciences of the United States of America 82, no. 16: 5490–5494. 10.1073/pnas.82.16.5490. 2410927 PMC391148

[brb371207-bib-0024] Hotter, B. , S. Hoffmann , L. Ulm , C. Meisel , J. B. Fiebach , and A. Meisel . 2019. “IL‐6 Plasma Levels Correlate With Cerebral Perfusion Deficits and Infarct Sizes in Stroke Patients Without Associated Infections.” Frontiers in Neurology 10: 83. 10.3389/fneur.2019.00083. 30828313 PMC6384225

[brb371207-bib-0025] Hozo, S. P. , B. Djulbegovic , and I. Hozo . 2005. “Estimating the Mean and Variance From the Median, Range, and the Size of a Sample.” BMC Medical Research Methodology 5: 13. 10.1186/1471-2288-5-13. 15840177 PMC1097734

[brb371207-bib-0026] Jara, M. D. J. , A. S. Gautam , V. Peesapati , M. Sadik , and S. Khan . 2020. “The Role of Interleukin‐6 and Inflammatory Cytokines in Pancreatic Cancer‐Associated Depression.” Cureus 12, no. 8: e9969. 10.7759/cureus.9969. 32850269 PMC7444958

[brb371207-bib-0027] Jiao, J. T. , C. Cheng , Y. J. Ma , et al. 2016. “Association Between Inflammatory Cytokines and the Risk of Post‐Stroke Depression, and the Effect of Depression on Outcomes of Patients With Ischemic Stroke in a 2‐Year Prospective Study.” Experimental and Therapeutic Medicine 12, no. 3: 1591–1598. 10.3892/etm.2016.3494. 27588080 PMC4998048

[brb371207-bib-0028] Kang, H. J. , K. Y. Bae , S. W. Kim , et al. 2016. “Effects of Interleukin‐6, Interleukin‐18, and Statin Use, Evaluated at Acute Stroke, on Post‐Stroke Depression During 1‐Year Follow‐Up.” Psychoneuroendocrinology 72: 156–160. 10.1016/j.psyneuen.2016.07.001. 27428088

[brb371207-bib-0029] Kang, Y. , Y. Yang , J. Wang , Y. Ma , H. Cheng , and D. Wan . 2021. “Correlation Between Intestinal Flora and Serum Inflammatory Factors in Post‐Stroke Depression in Ischemic Stroke.” Journal of the College of Physicians and Surgeons Pakistan 31, no. 10: 1224–1227. 10.29271/jcpsp.2021.10.1224. 34601846

[brb371207-bib-0030] Khan, A. W. , M. Farooq , M. J. Hwang , M. Haseeb , and S. Choi . 2023. “Autoimmune Neuroinflammatory Diseases: Role of Interleukins.” International Journal of Molecular Sciences 24, no. 9: 7960. 10.3390/ijms24097960. 37175665 PMC10178921

[brb371207-bib-0031] Köhler, C. A. , T. H. Freitas , M. Maes , et al. 2017. “Peripheral Cytokine and Chemokine Alterations in Depression: A Meta‐Analysis of 82 Studies.” Acta Psychiatrica Scandinavica 135, no. 5: 373–387. 10.1111/acps.12698. 28122130

[brb371207-bib-0032] Korostynski, M. , D. Hoinkis , M. Piechota , et al. 2021. “Toll‐Like Receptor 4‐Mediated Cytokine Synthesis and Post‐Stroke Depressive Symptoms.” Translational Psychiatry 11, no. 1: 246. 10.1038/s41398-021-01359-x. 33903586 PMC8076201

[brb371207-bib-0033] Lanctôt, K. L. , M. P. Lindsay , E. E. Smith , et al. 2020. “Canadian Stroke Best Practice Recommendations: Mood, Cognition and Fatigue Following Stroke, 6th Edition Update 2019.” International Journal of Stroke 15, no. 6: 668–688. 10.1177/1747493019847334. 31221036

[brb371207-bib-0034] Li, G. , J. Miao , C. Pan , et al. 2021. “Higher Serum Lactic Dehydrogenase Is Associated With Post‐Stroke Depression at Discharge.” Clinical Interventions in Aging 16: 2047–2055. 10.2147/CIA.S341169. 34916787 PMC8668225

[brb371207-bib-0035] Li, G. , J. Miao , W. Sun , et al. 2020. “Lower Serum Uric Acid Is Associated With Post‐Stroke Depression at Discharge.” Frontiers in Psychiatry 11: 52. 10.3389/fpsyt.2020.00052. 32132938 PMC7040095

[brb371207-bib-0037] Li, X. , W. Jin , L. Han , X. Chen , and L. Li . 2025. “Comparison and Application of Depression Screening Tools for Adolescents: Scale Selection and Clinical Practice.” Child and Adolescent Psychiatry and Mental Health 19, no. 1: 53. 10.1186/s13034-025-00908-2. 40346636 PMC12065149

[brb371207-bib-0038] Li, Y. , Y. Xie , Y. Xu , et al. 2022. “Interleukin‐6‐White Matter Network Differences Explained the Susceptibility to Depression After Stressful Life Events.” Journal of Affective Disorders 305: 122–132. 10.1016/j.jad.2022.03.003. 35271870

[brb371207-bib-0039] Liberati, A. , D. G. Altman , J. Tetzlaff , et al. 2009. “The PRISMA Statement for Reporting Systematic Reviews and Meta‐Analyses of Studies That Evaluate Health Care Interventions: Explanation and Elaboration.” Journal of Clinical Epidemiology 62, no. 10: e1–34. 10.1016/j.jclinepi.2009.06.006. 19631507

[brb371207-bib-0040] Lim, J. S. , J. J. Lee , and C. W. Woo . 2021. “Post‐Stroke Cognitive Impairment: Pathophysiological Insights Into Brain Disconnectome From Advanced Neuroimaging Analysis Techniques.” Journal of Stroke 23, no. 3: 297–311. 10.5853/jos.2021.02376. 34649376 PMC8521255

[brb371207-bib-0041] Liu, F. , L. Gong , H. Zhao , Y. L. Li , Z. Yan , and J. Mu . 2024. “Validity of Evaluation Scales for Post‐Stroke Depression: A Systematic Review and Meta‐Analysis.” BMC Neurology 24, no. 1: 286. 10.1186/s12883-024-03744-7. 39148052 PMC11325659

[brb371207-bib-0043] Liu, R. , L. Liu , S. Ren , et al. 2023. “The Role of IL‐33 in Depression: A Systematic Review and Meta‐Analysis.” Frontiers in Psychiatry 14: 1242367. 10.3389/fpsyt.2023.1242367. 38025419 PMC10646299

[brb371207-bib-0044] Lu, X. , J. Duan , Q. Cheng , and J. Lu . 2020. “The Association Between Serum Growth Differentiation Factor‐15 and 3‐Month Depression After Acute Ischemic Stroke.” Journal of Affective Disorders 260: 695–702. 10.1016/j.jad.2019.09.037. 31561112

[brb371207-bib-0045] Martínez‐Sánchez, P. , M. Gutiérrez‐Fernández , B. Fuentes , et al. 2014. “Biochemical and Inflammatory Biomarkers in Ischemic Stroke: Translational Study Between Humans and Two Experimental Rat Models.” Journal of Translational Medicine 12: 220. 10.1186/s12967-014-0220-3. 25086655 PMC4132215

[brb371207-bib-0046] Meng, G. , X. Ma , L. Li , et al. 2017. “Predictors of Early‐Onset Post‐Ischemic Stroke Depression: A Cross‐Sectional Study.” BMC Neurology 17, no. 1: 199. 10.1186/s12883-017-0980-5. 29149884 PMC5693521

[brb371207-bib-0047] Mohammed, S. , J. Haidar , B. A. Ayele , and Y. M. Yifru . 2023. “Post‐Stroke Limitations in Daily Activities: Experience From a Tertiary Care Hospital in Ethiopia.” BMC Neurology 23, no. 1: 364. 10.1186/s12883-023-03419-9. 37814255 PMC10561502

[brb371207-bib-0048] Monje, F. J. , M. Cabatic , I. Divisch , et al. 2011. “Constant Darkness Induces IL‐6‐Dependent Depression‐Like Behavior Through the NF‐κB Signaling Pathway.” Journal of Neuroscience 31, no. 25: 9075–9083. 10.1523/JNEUROSCI.1537-11.2011. 21697358 PMC6623479

[brb371207-bib-0049] Monsour, M. , D. M. Croci , S. Agazzi , and C. V. Borlongan . 2023. “Contemplating IL‐6, a Double‐Edged Sword Cytokine: Which Side to Use for Stroke Pathology.” CNS Neuroscience & Therapeutics 29, no. 2: 493–497. 10.1111/cns.14041. 36478506 PMC9873516

[brb371207-bib-0050] Morieri, M. L. , A. Passaro , and G. Zuliani . 2017. ““Interleukin‐6” Trans‐Signaling and Ischemic Vascular Disease: The Important Role of Soluble gp130.” Mediators of Inflammation 2017: 1396398. 10.1155/2017/1396398. 28250574 PMC5307001

[brb371207-bib-0051] Mu, Y. , Z. Wang , J. Zhou , C. Tan , and H. Wang . 2018. “Correlations of Post‐Stroke Depression With Inflammatory Response Factors.” Iranian Journal of Public Health 47, no. 7: 988–993. 30181997 PMC6119581

[brb371207-bib-0052] Nagy, E. E. , A. Frigy , J. A. Szász , and E. Horváth . 2020. “Neuroinflammation and Microglia/Macrophage Phenotype Modulate the Molecular Background of Post‐Stroke Depression: A Literature Review.” Experimental and Therapeutic Medicine 20, no. 3: 2510–2523. 10.3892/etm.2020.8933. 32765743 PMC7401670

[brb371207-bib-0053] Neupane, S. P. , A. Virtej , L. E. Myhren , and V. H. Bull . 2022. “Biomarkers Common for Inflammatory Periodontal Disease and Depression: A Systematic Review.” Brain, Behavior, & Immunity ‐ Health 21: 100450. 10.1016/j.bbih.2022.100450. PMC8938251 35330865

[brb371207-bib-0054] Ormstad, H. , H. C. Aass , K. F. Amthor , N. Lund‐Sørensen , and L. Sandvik . 2012. “Serum Levels of Cytokines, Glucose, and Hemoglobin as Possible Predictors of Poststroke Depression, and Association With Poststroke Fatigue.” International Journal of Neuroscience 122, no. 11: 682–690. 10.3109/00207454.2012.709892. 22812657

[brb371207-bib-0055] Qin, Y. , N. Wang , X. Zhang , X. Han , X. Zhai , and Y. Lu . 2018. “IDO and TDO as a Potential Therapeutic Target in Different Types of Depression.” Metabolic Brain Disease 33, no. 6: 1787–1800. 10.1007/s11011-018-0290-7. 30014175

[brb371207-bib-0056] Rose‐John, S. 2017. “The Soluble Interleukin‐6 Receptor: Advanced Therapeutic Options in Inflammation.” Clinical Pharmacology and Therapeutics 102, no. 4: 591–598. 10.1002/cpt.782. 28675418

[brb371207-bib-0057] Rothaug, M. , C. Becker‐Pauly , and S. Rose‐John . 2016. “The Role of Interleukin‐6 Signaling in Nervous Tissue.” Biochimica et Biophysica Acta 1863, no. 6: 1218–1227. 10.1016/j.bbamcr.2016.03.018. 27016501

[brb371207-bib-0058] Shi, Q. , R. Li , Z. Qu , et al. 2023. “Longitudinal Change of Six Common Inflammatory Cytokines and Their Relationship to Anxiety, Depression, and Cognitive Impairment in Acute Ischemic Stroke Patients.” Brazilian Journal of Medical and Biological Research 56: e13025. 10.1590/1414-431x2023e13025. 37878890 PMC10591487

[brb371207-bib-0059] Spalletta, G. , L. Cravello , F. Imperiale , et al. 2013. “Neuropsychiatric Symptoms and Interleukin‐6 Serum Levels in Acute Stroke.” Journal of Neuropsychiatry and Clinical Neurosciences 25, no. 4: 255–263. 10.1176/appi.neuropsych.12120399. 24247852

[brb371207-bib-0060] Su, J. A. , S. Y. Chou , C. S. Tsai , and T. H. Hung . 2012. “Cytokine Changes in the Pathophysiology of Poststroke Depression.” General Hospital Psychiatry 34, no. 1: 35–39. 10.1016/j.genhosppsych.2011.09.020. 22055333

[brb371207-bib-0061] Ting, E. Y. , A. C. Yang , and S. J. Tsai . 2020. “Role of Interleukin‐6 in Depressive Disorder.” International Journal of Molecular Sciences 21, no. 6: 2194. 10.3390/ijms21062194. 32235786 PMC7139933

[brb371207-bib-0062] Uciechowski, P. , and W. Dempke . 2020. “Interleukin‐6: A Masterplayer in the Cytokine Network.” Oncology 98, no. 3: 131–137. 10.1159/000505099. 31958792

[brb371207-bib-0063] Vignoli, A. , L. Tenori , C. Morsiani , P. Turano , M. Capri , and C. Luchinat . 2022. “Serum or Plasma (and Which Plasma), That Is the Question.” Journal of Proteome Research 21, no. 4: 1061–1072. 10.1021/acs.jproteome.1c00935. 35271285 PMC8981325

[brb371207-bib-0064] Vöckel, J. , A. Markser , L. Wege , H. L. Wunram , C. Sigrist , and J. Koenig . 2024. “Pharmacological Anti‐Inflammatory Treatment in Children and Adolescents With Depressive Symptoms: A Systematic‐Review and Meta‐Analysis.” European Neuropsychopharmacology 78: 16–29. 10.1016/j.euroneuro.2023.09.006. 37864981

[brb371207-bib-0065] Wang, L. , C. Chunyou , J. Zhu , X. Bao , and X. Tao . 2023. “Prediction of Post‐Stroke Depression With Combined Blood Biomarkers IL‐6, TNF‐α, and Fatty Acid Binding Protein: A Prospective Study.” Journal of Medical Biochemistry 42, no. 4: 638–644. 10.5937/jomb0-43904. 38084247 PMC10710798

[brb371207-bib-0066] Wang, P. , Y. B. Feng , L. Wang , et al. 2019. “Interleukin‐6: Its Role and Mechanisms in Rescuing Depression‐Like Behaviors in Rat Models of Depression.” Brain, Behavior, and Immunity 82: 106–121. 10.1016/j.bbi.2019.08.002. 31394209

[brb371207-bib-0067] Wang, Q. , Z. Zhu , Y. Liu , X. Tu , and J. He . 2018. “Relationship Between Serum Vitamin D Levels and Inflammatory Markers in Acute Stroke Patients.” Brain and Behavior 8, no. 2: e00885. 10.1002/brb3.885. 29484258 PMC5822590

[brb371207-bib-0068] Wang, Y. , W. Sun , J. Miao , et al. 2021. “Higher Fasting C‐Peptide Is Associated With Post‐Stroke Depression: A Multicenter Prospective Cohort Study.” BMC Neurology 21, no. 1: 383. 10.1186/s12883-021-02413-3. 34607565 PMC8489065

[brb371207-bib-0069] Wang, Y. , L. Zhu , X. Tan , Y. Cheng , X. Wang , and S. Fang . 2022. “Higher Levels of Peripheral Blood Glucose in the Acute Stage of Stroke Increase the Risk of Post‐Stroke Depression: A Systematic Review and Meta‐Analysis.” Neuroscience and Biobehavioral Reviews 142: 104829. 10.1016/j.neubiorev.2022.104829. 35970415

[brb371207-bib-0070] Wen, H. , K. B. Weymann , L. Wood , and Q. M. Wang . 2018. “Inflammatory Signaling in Post‐Stroke Fatigue and Depression.” European Neurology 80, no. 3–4: 138–148. 10.1159/000494988. 30448848

[brb371207-bib-0071] Wen, L. , C. Yan , T. Si , et al. 2024. “The Predictive Role of Early Inflammation and Oxidative Stress and the Dynamics of Cytokines Networks in Post‐Stroke Depression.” Journal of Affective Disorders 347: 469–476. 10.1016/j.jad.2023.12.012. 38065474

[brb371207-bib-0072] Xu, T. , S. Pu , Y. Ni , M. Gao , X. Li , and X. Zeng . 2018. “Elevated Plasma Macrophage Migration Inhibitor Factor as a Risk Factor for the Development of Post‐Stroke Depression in Ischemic Stroke.” Journal of Neuroimmunology 320: 58–63. 10.1016/j.jneuroim.2018.04.003. 29759141

[brb371207-bib-0073] Yang, L. , Z. Zhang , D. Sun , Z. Xu , X. Zhang , and L. Li . 2010. “The Serum Interleukin‐18 Is a Potential Marker for Development of Post‐Stroke Depression.” Neurological Research 32, no. 4: 340–346. 10.1179/016164110x12656393665080. 20482998

[brb371207-bib-0074] Yi, J. , C. Kim , and C. A. Gelfand . 2007. “Inhibition of Intrinsic Proteolytic Activities Moderates Preanalytical Variability and Instability of Human Plasma.” Journal of Proteome Research 6, no. 5: 1768–1781. 10.1021/pr060550h. 17411080

[brb371207-bib-0075] Young, J. J. , D. Bruno , and N. Pomara . 2014. “A Review of the Relationship Between Proinflammatory Cytokines and Major Depressive Disorder.” Journal of Affective Disorders 169: 15–20. 10.1016/j.jad.2014.07.032. 25128861

[brb371207-bib-0076] Yui, S. , D. Sasayama , M. Yamaguchi , and S. Washizuka . 2022. “Altered Levels of Salivary Cytokines in Patients With Major Depressive Disorder.” Clinical Neurology and Neurosurgery 221: 107390. 10.1016/j.clineuro.2022.107390. 35917728

[brb371207-bib-0077] Zemed, A. , K. Sany , and M. Gahaw . 2021. “Burden of Depression and Predictors Among Ethiopian Stroke Survivors: Cross‐Sectional Study.” Annals of Medicine and Surgery 71: 102926. 10.1016/j.amsu.2021.102926. 34712476 PMC8531554

[brb371207-bib-0078] Zhang, X. F. , W. Zou , and Y. Yang . 2016. “Effects of IL‐6 and Cortisol Fluctuations in Post‐Stroke Depression.” Journal of Huazhong University of Science and Technology [Medical Sciences] 36, no. 5: 732–735. 10.1007/s11596-016-1653-0. 27752894

[brb371207-bib-0079] Zheng, T. , T. Jiang , R. Li , Y. Zhu , Q. Han , and M. Wang . 2024. “Circulating Interleukins Concentrations and Post‐Stroke Depression: A Systematic Review and Meta‐Analysis.” Progress in Neuro‐Psychopharmacology & Biological Psychiatry 134: 111050. 10.1016/j.pnpbp.2024.111050. 38844127

[brb371207-bib-0080] Zhou, H. , Y. J. Wei , and G. Y. Xie . 2024. “Research Progress on Post‐Stroke Depression.” Experimental Neurology 373: 114660. 10.1016/j.expneurol.2023.114660. 38141804

